# Integrated Microbiota and Metabolite Changes following Rice Bran Intake during Murine Inflammatory Colitis-Associated Colon Cancer and in Colorectal Cancer Survivors

**DOI:** 10.3390/cancers15082231

**Published:** 2023-04-10

**Authors:** Annika M. Weber, Hend Ibrahim, Bridget A. Baxter, Robin Kumar, Akhilendra K. Maurya, Dileep Kumar, Rajesh Agarwal, Komal Raina, Elizabeth P. Ryan

**Affiliations:** 1 Department of Food Science and Human Nutrition, Colorado State University, Fort Collins, CO 80523, USA; 2 Department of Medical Biochemistry, Faculty of Medicine, Zagazig University, Zagazig 44519, Egypt; 3 Department of Environmental and Radiological Health Sciences, Colorado State University, Fort Collins, CO 80523, USA; 4 Department of Pharmaceutical Sciences, South Dakota State University, Brookings, SD 57007, USA; 5 Department of Pharmaceutical Sciences, University of Colorado Denver—Anschutz Medical Campus, Aurora, CO 80045, USA

**Keywords:** AOM/DSS, rice bran, microbiome, metabolome, colorectal cancer

## Abstract

**Simple Summary:**

Heat-stabilized rice bran is a nutrient-dense food ingredient that has shown colorectal cancer control and prevention properties in a suite of carcinogen-induced animal models. This study identified colon cancer protective properties associated with rice bran metabolism in feces that relate to the previously reported findings in gastrointestinal tissue. A longitudinal analysis of fecal microbiota and metabolite changes between diet treatments identified novel mechanisms for rice bran-mediated anti-cancer activity. Fecal metabolites in response to dietary rice bran intake in mice showed overlap with results from adult colorectal cancer survivors after eating rice bran. These molecules may serve as translational dietary biomarkers for colorectal cancer prevention.

**Abstract:**

Dietary rice bran-mediated inhibition of colon carcinogenesis was demonstrated previously for carcinogen-induced rodent models via multiple anti-cancer mechanisms. This study investigated the role of dietary rice bran-mediated changes to fecal microbiota and metabolites over the time course of colon carcinogenesis and compared murine fecal metabolites to human stool metabolic profiles following rice bran consumption by colorectal cancer survivors (NCT01929122). Forty adult male BALB/c mice were subjected to azoxymethane (AOM)/dextran sodium sulfate (DSS)-induced colitis-associated colon carcinogenesis and randomized to control AIN93M (*n* = 20) or diets containing 10% *w*/*w* heat-stabilized rice bran (*n* = 20). Feces were serially collected for 16S rRNA amplicon sequencing and non-targeted metabolomics. Fecal microbiota richness and diversity was increased in mice and humans with dietary rice bran treatment. Key drivers of differential bacterial abundances from rice bran intake in mice included *Akkermansia*, *Lactococcus*, *Lachnospiraceae*, and *Eubacterium xylanophilum*. Murine fecal metabolomics revealed 592 biochemical identities with notable changes to fatty acids, phenolics, and vitamins. Monoacylglycerols, dihydroferulate, 2-hydroxyhippurate (salicylurate), ferulic acid 4-sulfate, and vitamin B6 and E isomers significantly differed between rice bran- and control-fed mice. The kinetics of murine metabolic changes by the host and gut microbiome following rice bran consumption complemented changes observed in humans for apigenin, N-acetylhistamine, and ethylmalonate in feces. Increased enterolactone abundance is a novel diet-driven microbial metabolite fecal biomarker following rice bran consumption in mice and humans from this study. Dietary rice bran bioactivity via gut microbiome metabolism in mice and humans contributes to protection against colorectal cancer. The findings from this study provide compelling support for rice bran in clinical and public health guidelines for colorectal cancer prevention and control.

## 1. Introduction

Colorectal cancer (CRC) rates are increasing in adults under the age of 50 and it is the second leading cause of death from cancer [1]. Adopting healthy lifestyle changes is critical to CRC control and prevention [2]. The number of deaths related to CRC is expected to rise, and the high burden of CRC deserves urgent attention through affordable and accessible strategies. Diet is one major lifestyle factor strongly associated with CRC risk and development [3,4,5]. In particular, dietary intakes of fiber, fatty acids, micronutrients, and phytochemicals from whole grains and legumes and fruits and vegetables have been shown to drastically modulate CRC risk [6,7,8,9]. 

Whole grain brown rice and rice bran contain unique bioactive phytochemicals, vitamins, and prebiotics that have exhibited the control and prevention of several types of human cancers [10,11,12,13], including colorectal cancer in both animals and humans [14,15,16,17,18,19]. Rice bran is the nutrient-rich outer layer of rice milled off to yield white rice. Rice bran metabolism by intestinal microbiota promotes intestinal barrier function and mucosal immunity and modulates gut microflora community composition [19,20,21,22,23,24]. Rice bran CRC protective phytochemicals induce apoptosis and block cell proliferation to prevent tumor growth [25,26,27]. Numerous studies have previously documented the CRC prevention properties associated with brown rice and rice bran [13,14,16,17,28,29,30]. The bioactive metabolites from rice bran reduce inflammation by scavenging free radicals [31] and triggering anti-tumor immune responses in the colonic microenvironment [32,33,34]. 

The role of the gut microbiome in CRC has gained increasing attention with relationships between inflammation, anti-tumor immunity, and cell growth signaling pathways [35]. The association between gut microbiota and diet components for disease prevention includes the production of bacteria-derived metabolites [36]. Commensal microbiota metabolites, such as the generation of short-chain fatty acids (SCFAs), play roles in immune regulation by reducing inflammation and cancer outcomes [37,38,39]. Studies have also shown correlations between gut microbiota diversity and CRC development. Some CRC patients have dysbiosis characterized by elevated taxa such as *Fusobacteria* and *Firmicutes* [40,41,42,43,44]. Globally, gut microbiome function is imperative to understanding dietary mechanisms for the control and prevention of CRC. 

Though there are well-established studies in the literature documenting rice bran’s role in CRC control and prevention [13,14,16,17,28,29,30], a gap in knowledge exists for the time-dependent, weekly changes to fecal microbial communities and metabolites associated with the host digestion of rice bran, which are directly and indirectly contributing to reducing CRC formation. In this regard, we recently reported how rice bran intake results in less local and systemic inflammation in the colon tissue and plasma in an AOM-DSS pre-clinical murine model [17]. The rice bran-fed mouse group had enriched *Clostridiales*, *Lachnospiraceae*, and *Ruminiclostridium* in the cecum and colon. Metabolites such as hippurate, ferulic acid 4-sulfate, and trigonelline were also elevated in the colon tissue of the mice. Notably, the cecum and colonic tissue metabolite and microbiota differences were revealed at week 14, and that study did not elucidate time-dependent changes in the feces. Based on compelling findings on protection in colon tissue, the primary objective of this study was to examine and establish fecal microbiota and metabolite modifications following rice bran consumption that occur during 2–14 weeks of colon carcinogenesis that contributed to CRC protection at 14 weeks. The strong relationship between murine fecal metabolite changes was directly compared to human stools after rice bran intake for practical and translational non-invasive monitoring of diet-mediated reductions in colorectal cancer. 

## 2. Materials and Methods

### 2.1. Rice Bran and Control Diet Preparation 

Ri300 heat-stabilized rice bran used in the animal diets was purchased from Rice Bran Technologies (Sacramento, CA, USA). Rice bran stabilization took place at 110 °C for 30 min in a commercial dryer as described previously [45]. Mouse diets were prepared as previously described using AIN-93M purified components (Envigo, Madison, WI, USA) as the control diet [16]. The control diet was composed of 4% *w*/*v* corn oil, casein, L-cystine, corn starch, maltodextrin, sucrose, cellulose, mineral and vitamin mix, choline bitartrate, and tertiary butyl-hydroquinone antioxidant. The 10% *w*/*w* heat-stabilized rice bran diet was adjusted across ingredients to account for nutrients supplied by the rice bran [46,47]. 

### 2.2. Ethics Statement

Animal experiments were performed at The University of Colorado Denver-AMC animal facility in alignment with the approved Institutional Animal Care and Use Committee (IACUC) protocol and an Inter-Institutional Agreement with Colorado State University. All methods carried out were in accordance with these guidelines and the ARRIVE guidelines. 

### 2.3. Animal Study Design and Sample Collection

**Figure 1:**
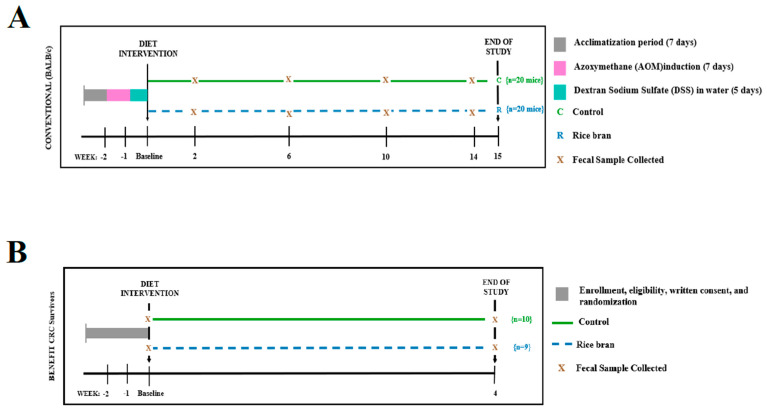
Experimental design for rice bran intervention in mice and humans for CRC control and prevention. (**A**) Six-week-old Balb/c mice were fed an AIN-93M pellet diet and acclimatized for one week. All mice then received a single intraperitoneal injection of 10 mg/kg body weight of azoxymethane (AOM) in saline. Seven days after AOM injection, mice were subjected to 2% dextran sodium sulfate (DSS) in drinking water for five days. Mice were then randomized and switched to either the rice bran (*n* = 20) diet group or maintained on the control AIN-93M pellet (*n* = 20) diet. Feces were collected from mice in both groups at baseline, and 2, 6, 10, and 14 weeks. (**B**) Human dietary rice bran intervention trial in CRC survivors (NCT01929122). Participants were provided daily meals and snacks with rice bran (*n* = 9) or no rice bran (*n* = 10) for 4 weeks. Stool samples were collected at baseline and week 4.

The methods of this study have been previously published [17]. Six-week-old male BALB/c mice (*n* = 40) (Charles River Laboratories, Wilmington, MA, USA) were housed in an animal facility at The University of Colorado Denver-AMC, fed a control AIN-93M pellet diet, and acclimatized for 1 week. A single intraperitoneal injection of 10 mg/kg body weight of azoxymethane (AOM) (Millipore Sigma, Temecula, CA, USA) in saline was administered to all mice. Seven days later, a 5-day cycle of 2% dextran sodium sulfate (DSS) (mol. wt. 36,000–50,000; MP Biomedicals, LLC, Santa Ana, CA, USA) was replaced in drinking water. After DSS exposure, mice were randomized and switched to either a supplemented rice bran diet (*n* = 20) or maintained on the control AIN-93M pellet diet (*n* = 20) for a 14-week period. Body weight, general health, and food consumption by the mice were recorded weekly over the course of the study. During the weeks following AOM injection, fecal samples were collected as a function of time from mice in both groups at the following points: day 2 (baseline), and weeks 2, 6, 10, and 14. The study design, AOM treatment, cycles of DSS, and murine sample collection are depicted in (Figure 1A). Fecal samples were pooled from all mice within a single cage (*n* = 5 mice per cage) and immediately stored frozen at −80 °C. At week 15, mice were subjected to CO_2_ asphyxiation and euthanized by exsanguination. Further details of this have been previously published [17].

### 2.4. Metataxonomics Sample Processing, Sequencing, and Analysis

DNA extraction for the microbiome analysis from the fecal samples was processed using previously published methods [47]. Briefly, lyophilized fecal samples were homogenized and 50 mg/sample was used for DNA extraction with the MoBio PowerSoil Kit (MoBio Laboratories Inc., Solana Beach, CA, USA). Extracted DNA samples were stored at −20 °C until concentration and quality checking on a NanoDrop 2000 (Thermo Fisher Scientific, USA). V4 hypervariable region amplification of the 16S rRNA gene and amplicon sequencing on an Illumina MiSeq platform (Illumina Inc., San Diego, CA, USA) observed guidelines by the Earth Microbiome Project [48,49] and utilized the 515F/806R (Parada/Apprill) primer pair. Raw, single-end FASTQ forward sequence reads were analyzed using QIIME2 [47]. Feature tables of absolute relative abundance amplicon sequence variants (ASV) were constructed for each sample using reads from the DADA2 pipeline [50]. Taxonomic identities for ASV representative sequences were assigned with Naive Bayes classifiers independently trained on 99% OTU reference collections bound by the 515F/806R (Parada/Apprill) primer pair and trimmed to 248bp extracted from either the Greengenes 13_8 [51] or SILVA 132 [52] marker gene databases. Data were exported from QIIME2 to R for further analysis [53].

### 2.5. Computation Detail for Microbiome Analysis

Reads were analyzed using QIIME2 2019.10 in the Nextflow-core ampliseq pipeline (version 1.1.2, https://doi.org/10.5281/zenodo.3585924, accessed on 17 July 2020) using Singularity containerization. This pipeline uses DADA2 to generate ASVs, additional filtering (using “--min_frequency 2” and “--exclude_taxa “mitochondria,chloroplast”), machine learning taxonomy with a pre-trained SILVA reference dataset from QIIME2 (silva-132-99-515-806-nb-classifier.qza), generates alpha and beta diversity metrics, collectors curves, and PCoA plots, and carries out ANCOM and other statistical tests. ANCOM identified differentially abundant features employed here at the genus level between diet types, where W scores indicated the importance of the feature and center log ratio (clr) aggregated one feature against the community or average. 

### 2.6. Non-Targeted Metabolomics Sample Process

All fecal samples were sent to Metabolon Inc. (Durham, NC, USA) for non-targeted metabolite profiling via Waters ACQUITY ultra-performance liquid chromatography (UPLC; Waters, Milford, MA, USA) and a Thermo Fisher Scientific Q Exactive high-resolution/accurate mass spectrometer (UPLC-MS/MS) interfaced with a heated electrospray ionization (HESI-II) source and Orbitrap mass analyzer (Thermo Fisher Scientific, Waltham, MA) operated at a 35,000 mass resolution. The methods used have been previously described [16,17,47]. Briefly, samples were prepared using the automated MicroLab Star^®^ system (Hamilton Robotics Inc., Reno, NV, USA) Standards were added before the extraction process’s first step for quality control purposes. Several recovery standards were added before the extraction process’s first step for QC purposes. To remove protein, small molecules bound to protein or trapped in the precipitated protein matrix were dissociated, chemically diverse metabolites were recovered, and proteins were precipitated with methanol under vigorous shaking for 2 min (GenoGrinder 2000; Glen Mills Inc., Clifton, NJ, USA), followed by centrifugation. The resulting extract was divided into five fractions: two for analysis by two separate reverse phase (RP)/UPLC-MS/MS methods with positive ion mode electrospray ionization (ESI), one for analysis by RP/UPLC-MS/MS with negative ion mode ESI, one for analysis by HILIC/UPLC-MS/MS with negative ion mode ESI, and one sample was reserved for backup. Samples were placed briefly on a TurboVap^®^ (Zymark, Hopkinton, MA, USA) to remove the organic solvent. The sample extract was dried and then reconstituted in solvents compatible with each of the four methods. Each reconstitution solvent contained a series of standards at fixed concentrations to ensure injection and chromatographic consistency. One aliquot was analyzed using acidic positive ion conditions, chromatographically optimized for more hydrophilic compounds. In this method, the extract was gradient eluted from a C18 column (Waters UPLC BEH C18-2.1 × 100 mm, 1.7 µm) using water and methanol, containing 0.05% perfluoropentanoic acid (PFPA) and 0.1% formic acid (FA). Another aliquot was also analyzed using acidic positive ion conditions; however, it was chromatographically optimized for more hydrophobic compounds. In this method, the extract was gradient eluted from the same aforementioned C18 column, using methanol, acetonitrile, water, 0.05% PFPA, and 0.01% FA, and was operated at an overall higher organic content. Another aliquot was analyzed using basic negative ion optimized conditions using a separate dedicated C18 column. The basic extracts were gradient eluted from the column using methanol and water, however with 6.5 mM ammonium bicarbonate at pH 8. The fourth aliquot was analyzed via negative ionization following elution from an HILIC column (Waters UPLC BEH amide 2.1 × 150 mm, 1.7 µm; Waters, Milford, MA, USA), using a gradient consisting of water and acetonitrile with 10 mM ammonium formate, pH 10.8. The MS analysis alternated between MS and data-dependent MSn scans using dynamic exclusion. The scan range varied slightly between methods but covered 70–1000 *m*/*z*. The extraction of raw data, peak identification, and processing for quality control were performed using software from Metabolon. The hardware and software foundations for these informatics components were the LAN backbone, and a database server running Oracle 10.2.0.1 Enterprise Edition. Medians were then rescaled and set to one. 

### 2.7. Metabolomics Statistical Analysis

Metabolite raw abundances were normalized by dividing the median raw abundance of each metabolite across the entire dataset for each matrix, which also produced median-scaled abundances, as previously performed [47]. Where a metabolite was missing for a sample, the minimum median scale abundance of that metabolite in the entire dataset was used as a minimum value so that downstream statistics could be performed. Calculations for metabolite fold differences for each metabolite were the quotient of the average median-scaled abundance of the metabolite in the treatment group (rice bran) by that of the control group. Metabolites were compared using two-way ANOVA with a Welch’s post hoc test, with significance defined as *p* < 0.05. To account for false discovery rate errors, a q-value was calculated for each metabolite. Metabolite data visualization was graphed and visualized in GraphPad Prism 9.4.0. 

### 2.8. Human Study Design: Beans/Bran Enriching Nutritional Eating for Intestinal Health Trial (BENEFIT) 

The complete study design and primary outcomes from the Beans/Bran Enriching Nutritional Eating for Intestinal Health Trial (BENEFIT, NCT01929122) have been previously published [20,22,54,55]. A summary of this study design is depicted in Figure 1B. The study was conducted in accordance with the Declaration of Helsinki and all procedures involving human participants were approved by the Poudre Valley Hospital/University of Colorado Health-North Institutional Review Board (Protocol No. 10-1038) and Colorado State University Institutional Review Board (Protocol No. 09-1520H). Written informed consent was obtained from all participants for follow-up investigations. Briefly, nineteen CRC survivors recruited from the University of Colorado-North Cancer Center network participated in a parallel randomized controlled dietary interventional trial. Participants in the control arm (*n* = 10) were 60% female, aged 64 ± 14, and had an average BMI of 27.3 ± 3.3. In the rice bran arm (*n* = 9), participant demographics were 56% female, with an average age of 62 ± 8, and an average BMI of 28.7 ± 5.2. Participants were provided meals/snacks with either 30/g of rice bran (*n* = 9) or no rice bran (*n* = 10) for 4 weeks. All study foods were ~1/3 of the total energy content consumed daily by participants and the remaining 2/3 of daily energy content was not controlled, though it was recorded and analyzed. All food logs were tracked in Nutritionist Pro™ (Axxya Systems, Redmond, WA, USA). Participants provided self-collected stool samples. Stool microbiota and multi-matrix metabolite profiles were previously published [20,22,55]. 

### 2.9. Availability of Data and Materials

All data presented and analyzed in this investigation are included in this article. Public access for microbiome sequencing files was performed to ensure the transparency and complete reproducibility of the microbiome analysis performed herein. The amplicon sequence data supporting the results and conclusions of this manuscript are available via the NCBI SRA BioProject Accession No. PRJNA516457. Fecal metabolite profiles are available upon request.

## 3. Results

BALB/c mice were subjected to AOM/DSS-induced colitis as a model of inflammatory colon carcinogenesis. The study design, timeline of AOM treatment, cycles of DSS, rice bran dietary intervention, and murine sample collection are depicted in Figure 1A. We recently reported the protective effects of rice bran intake in this inflammation-associated colon cancer progression model [17], whereby a reduction in the size of neoplastic lesions and less mucosal inflammation in the colon tissue were reported alongside the restoration of colonic epithelial integrity. These histopathological findings and molecular marker assessments, as published in [17], are summarized in Table S1. In continuation of our previous observations, the present study was conducted to examine the fecal microbiome and metabolite changes during a 2–14-week timeframe of carcinogenesis and throughout the course of a rice bran inhibition of colon cancer. Longitudinal analysis of fecal microbiota and metabolic changes following rice bran intake has not been previously examined.

### 3.1. Rice Bran Mediated Changes to Fecal Microbiota Composition during Colon Carcinogenesis

**Figure 2:**
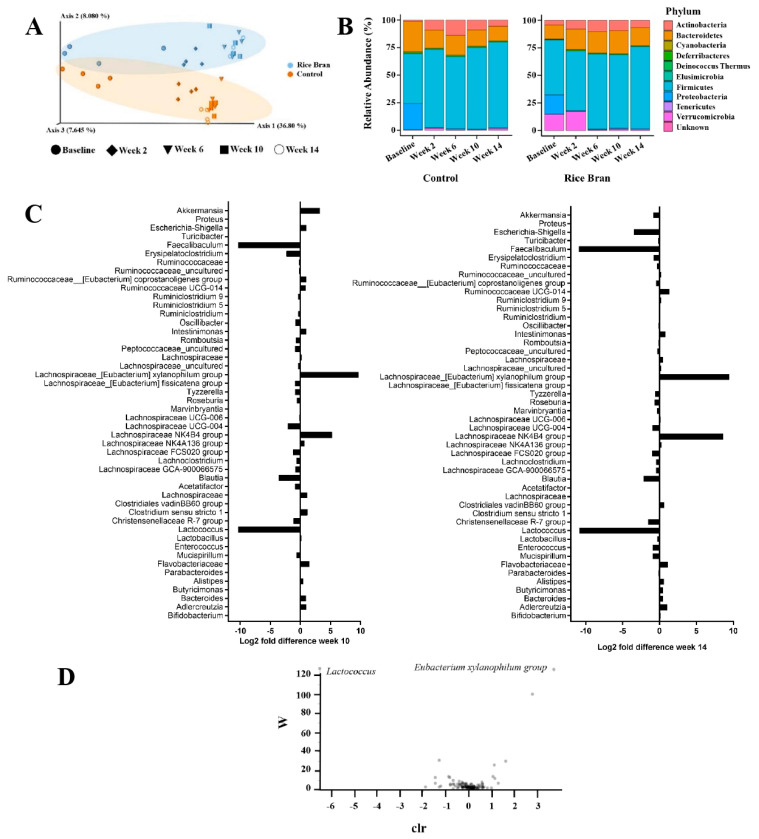
Time-dependent fecal microbiota changes following control and rice bran intake in AOM/DSS-treated mice. (**A**) Unweighted UniFrac PCoA plot of the fecal microbiome separation by diet: rice bran in blue, control in orange. (**B**) Phylum-level changes in rice bran and control groups at baseline, and 2, 6, 10, and 14 weeks. (**C**) Genus-level log2 fold differences in the rice bran group compared to the control at week 10 and week 14. (**D**) ANCOM volcano plots, centered log ratio (clr) with W test statistic, and differentially abundant features between diet types. Statistically significant features as calculated by ANCOM are labelled.

A significant difference in Observed OTU alpha diversity was detected between the diet types (Kruskal–Wallis, *p* = 0.025, Table S2). No significant difference was observed between the rice bran and control at baseline in any of the four alpha diversity measures by Kruskal–Wallis pairwise comparisons for Evenness, Faith Phylogenetic Diversity, Observed OTUs, or Shannon (Table S3). Significant differences in Bray Curtis, Weighted UniFrac Distance Matrix, Jaccard Distance, and Unweight UniFrac Distance Matrix were detected between diet groups (Table S2). Two beta diversity indices, Jaccard Distance and Unweighted UniFrac Distance Matrix, revealed no significant differences between groups at baseline, yet the Bray Curtis and Weighted UniFrac Distance Matrix indices did reveal differences (Table S3). Further measures of beta diversity are represented in Table S4 and Figure 2A, which demonstrates PERMANOVA pairwise comparisons of Unweighted UniFrac and PCoA plot. To understand the effects of rice bran on the species richness and species diversity of fecal microbiomes during colon carcinogenesis, we also analyzed non-phylogenetic alpha diversity metrics. Feces from mice in the rice bran treatment group had significantly different Observed OTU alpha diversity over time (Table S5 and Figure S1). There was no statistical difference in the OTUs of the rice bran and control groups at baseline (Table S3). There were statistical differences in the number of OTUs detected between the rice bran and control groups each week (weeks 2, 6, 10, and 14; Table S5).

Unweighted UniFrac PERMANOVA pairwise comparisons identified no significant difference between the rice bran and control OTUs at baseline (*p* > 0.05, Table S4). Starting at week 2, there was a significant difference (*p* = 0.031, Table S4) in the OTUs between the two groups. As seen in the PCoA plot, there were changes in the beta diversity of both groups each week. Each diet type was more similar to each other than to the other diets. This clear separation demonstrates the shift in OTU abundances according to diet group.

Ten known bacterial phyla were identified: *Actinobacteria*, *Bacteroidetes*, *Cyanobacteria*, *Deferribacteres*, *Deinococcus*, *Elusimicrobia*, *Firmicutes*, *Proteobacteria*, *Tenericutes*, and *Verrucomicrobia* (Figure 2B). Dominant phyla consisted of *Firmicutes*, *Bacteroidetes*, and *Actinobacteria*. Twenty-nine predominant genera in murine feces were detected in the rice bran and control. Changes in genus-level taxa at weeks 10 and 14 in the rice bran group compared to the controls are depicted in Figure 2C. In the rice bran group, decreased log2 fold differences at 10 weeks were in the genera *Lactococcus*, *Christensenellaceae R-7 group*, *Blautia*, *Lachnospiraceae FCS020 group*, *Lachnospiraceae UCG-004*, *Erysipelatoclostridium*, and *Faecalibaculum*. Increases in the rice bran group compared to the control at week 10 were seen in *Flavobacteriaceae_uncultured, Clostridium sensu stricto 1*, *Lachnospiraceae NK4B4 group*, *Eubacterium xylanophilum group*, and *Akkermansia.* At week 14, a decreased log2 fold difference in the rice bran group was seen in *Lactococcus*, *Christensenellaceae R-7 group*, *Blautia*, *Lachnospiraceae FCS020 group*, *Faecalibaculum*, and *Escherichia-Shigella.* An increased log2 fold difference at week 14 in the rice bran mice compared to the control was seen in *Adlercreutzia*, *Flavobacteriaceae*, *Lachnospiraceae NK4B4 group*, *Eubacterium xylanophilum group*, and *Ruminococcaceae UCG-014*. Genus-level changes at weeks 10 and 14 in the rice bran group compared to the control included *Flavobacteriaceae*, *Lactococcus*, *Christensenellaceae R-7 group*, *Blautia*, *Lachnospiraceae FCS020 group*, *Lachnospiraceae NK4B4 group*, *Eubacterium xylanophilum group*, and *Faecalibaculum*.

Analysis of the composition of microbiomes (ANCOM) was employed to identify drivers in differences between the fecal bacteria by diet type. Two differentially abundant features between diet types were identified to be significant by ANCOM. These key features included the *Eubacterium xylanophilum group,* which was statistically more abundant in the rice bran group, and *Lactococcus*, which was identified as statistically more abundant in the control group (Figure 2D).

### 3.2. Rice Bran Modulation of Fecal Metabolites for CRC Protection in Mice

**Figure 3:**
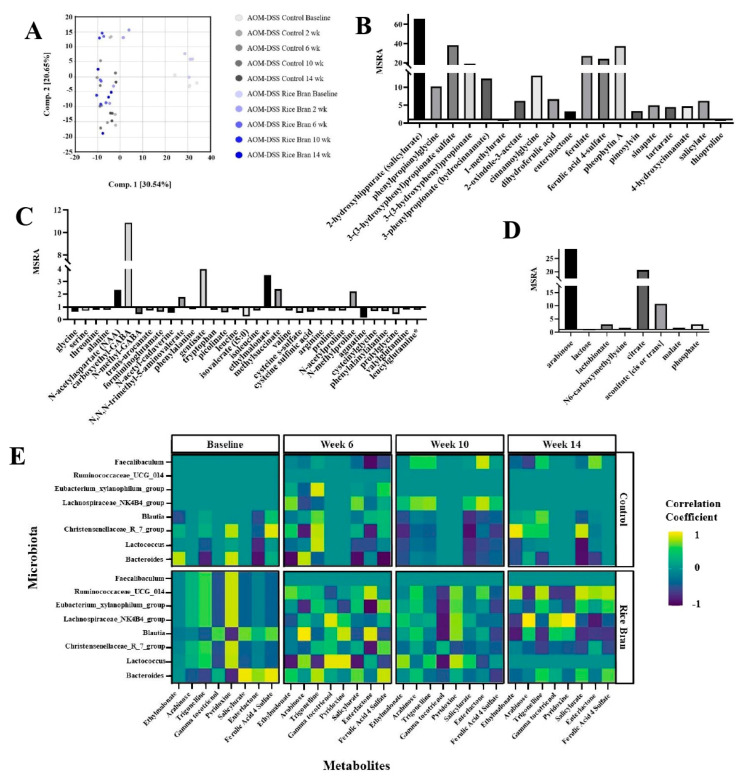
Fecal metabolite profiles modified by rice bran intake in AOM/DSS-treated mice. (**A**) PCA demonstrates segregation of the baseline to the 2- to 14-week time points. Stool metabolite mean relative scaled abundance (MSRA) fold difference with statistical significance between mice fed rice bran and the control at 14 weeks (*p* < 0.05) and clustered into chemical classifications as (**B**) phytochemicals, (**C**) amino acids and peptides, and (**D**) carbohydrates and energy. (**E**) Pearson correlation heatmap depicting selected gut microbiota and fecal metabolites in AOM/DSS mice at baseline, week 6, week 10, and week 14, for both the control and rice bran groups.

Fecal metabolite analysis revealed 702 biochemicals, 592 of which had known biochemical names. As shown in Figure 3A, principal component analysis (PCA) revealed segregation between the baseline (day 2) of rice bran and control samples compared to the rest of the time points. The PCA plot demonstrates the marked differences between the diet groups after baseline, suggesting metabolite differences at weeks 2, 6, 10, and 14. These results demonstrate the similar fecal metabolite profiles at baseline and the similarities in metabolite modulation by diet group after baseline. 

Table 1 lists a selected suite of metabolite changes at weeks 10 and 14 in the rice bran group compared to the control. These were selected based on having significant differences either at week 10 or week 14 (*p* < 0.05) and had a fold difference of >1.5 or <0.5 cut-off in either week 10, 14, or both, and with reported relevance to cancer progression or prevention. Tables S6 and S7 depict all metabolites at weeks 10 and 14 with a significant fold difference between the rice bran and control groups.

Cofactors and vitamins were increased in fecal abundance for the rice bran group at weeks 10 and 14. Notably, rice bran intake increased nicotinate (e.g., quinolinate, trigonelline), pantothenate, CoA (e.g., pantetheine), tocopherol (e.g., alpha-tocotrienol, gamma-tocotrienol), vitamin A (e.g., carotene diol), thiamin (e.g., thiamin), and vitamin B6 (e.g., pyridoxine). Vitamin B6 (pyridoxine) had a 41.89-fold increase in the rice bran group compared to the control at 14 weeks (Table 1). Furthermore, the multitude of phytochemicals that increased in the rice bran group included 2-hydroxyhippurate (salicylurate), 3-(3-hydroxyphenyl)propionate, 3-phenylpropionate, enterolactone, ferulate, dihydroferulic acid, pinosylvin, pheophytin A, sinapate, and salicylate (Figure 3B).

Amino acids with a significant fold difference in the rice bran group compared to the control at week 10 included carboxyethyl-GABA, N,N,N-trimethyl-5-aminovalerate, and N-formylmethionine. At 14 weeks, carboxyethyl-GABA and N,N,N-trimethyl-5-aminovalerate significantly differed (10.85- and 1.80-fold increases in the rice bran group, respectively), as is also depicted in Figure 3C. 

Regarding peptide metabolites, gamma-glutamyl-histidine had a significant fold increase in the rice bran to control group at week 10, and all other peptide-related metabolites had a significant fold decrease in the rice bran to control group at week 10 and week 14. There was also a 9.61- and 34.58-fold increase in arabinose in the carbohydrate chemical class in the rice bran group mice at weeks 10 and 14, respectively, compared to the control (Table 1 and Figure 3D). Changes in energy metabolism in the rice bran group at weeks 10 and 14 included an increase in fold change in TCA cycle metabolites: citrate (6.70- and 20.56-fold increases, respectively) and malate (1.54- and 1.74-fold increases, respectively), and at week 14, arabinose increased by ~35-fold in the mice consuming rice bran verses control. 

As depicted in Table 1, altered lipid metabolism was evidenced by decreases in SCFA (e.g., valerate (5:0)), medium-chain fatty acids (e.g., caproate (6:0)), and long-chain fatty acids (e.g., eicosenoate (20:1)) in the rice bran group at weeks 10 and 14. Furthermore, polyunsaturated fatty acids such as linoleate (18:2n6) and linoleate alpha or gamma (18:3n3 or 6) were higher in the rice bran group than in the controls. Metabolites such as glycerol 3-phosphate, monoacylglycerols, and diacylglycerols significantly increased in the rice bran group at 10 and 14 weeks. Lipid metabolites with fold increases above 12-fold and that had a statistically significant fold difference in the rice bran group included octadeccenedionate (C18:1–DC) (12.04-fold) and 16-hydroxypalmitate (12.32-fold). 

A Pearson correlation coefficient analysis identified associations between fecal microbiota and metabolites over the 14-week period (Figure 3E). Pearson correlation demonstrated time-dependent modulation during cancer progression in the control mice and for cancer prevention in the rice bran-fed mice. At baseline, which was day 2 of the study, there were associations between microbiota and metabolites. *Bacteroides* was correlated with fecal metabolites such as salicylurate, enterolactone, and ferulic acid 4-sulfate at baseline in the rice bran group, and the strength of this association lessened over time. Microbial–metabolite correlations that had a positive correlation over time in the rice bran group were *Lachnospiraceae NK4B4 group* and pyridoxine and *Lachnospiraceae NK4B4 group* and gamma-tocotrienol (Table S8). There was also a positive correlation between *Ruminococcaceae UCG 014* and the metabolites salicylurate, enterolactone, and ferulic acid 4-sulfate that strengthened over time in the rice bran group. There was a persistent strong negative correlation between the control *Lactococcus* and salicylurate in control mice. *Faecalibaculum* showed limited associations with fecal metabolites in either the control or rice bran group mice. 

### 3.3. Rice Bran Diet Mediated Metabolite Changes in Humans and AOM/DSS Mice

**Figure 4:**
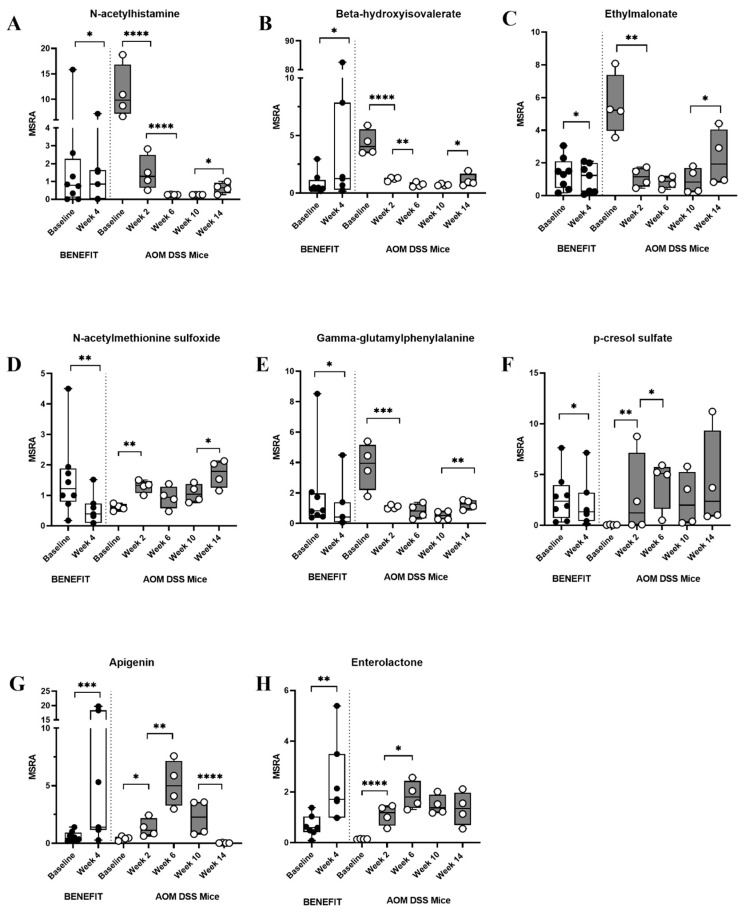
Median-scaled relative abundances (MSRA) for human fecal metabolites from the Beans/Bran Enriching Nutritional Eating For Intestinal Health Trial (BENEFIT) study at 4 weeks compared to the baseline are depicted (**left**). MSRAs for murine fecal metabolites from the rice bran group at baseline, and 2, 6, 10, and 14 weeks (**right**). (**A**) N-acetylhistamine, (**B**) beta-hydroxyisovalerate, (**C**) ethylmalonate, (**D**) N-acetylmethionine sulfoxide, (**E**) gamma-glutamylphenylalanine, (**F**) p-cresol sulfate, (**G**) apigenin, and (**H**) enterolactone (* = *p* < 0.05, ** = *p* < 0.01, *** = *p* < 0.001 and **** = *p* < 0.0001).

Metabolic changes related to rice bran consumption in humans (BENEFIT, NCT01929122) were analyzed for comparison with the mice. Human stool metabolite analysis revealed 39 significantly different metabolites in participants consuming rice bran compared to the controls [22]. Metabolic pathways of particular importance included fatty acids, vitamin B6, and amino acids (e.g., leucine and valine). Other human stool metabolites derived from rice bran consumption, such as 2-hydroxyhippurate (salicylurate), increased compared to the controls at week 4 [22]. Eight metabolites had a significant fold change in both the BENEFIT participants consuming rice bran (*p* < 0.05) and in at least two time points in the AOM/DSS rice bran group murine model (*p* < 0.05) (Table 2 and Table S9). N-acetylhistamine and ethylmalonate represent two stool metabolites with similar kinetics in the BENEFIT participants and AOM/DSS murine model over time (Figure 4A,C and Table S9). Apigenin and enterolactone increased in both BENEFIT participants and the present AOM/DSS mouse model over time (Figure 4G,H). N-acetylmethionine sulfoxide, gamma-glutamylphenylalanine, and p-cresol sulfate decreased in BENEFIT participants between baseline and week 4, whereas changes to these fecal metabolites in mice increased in the mouse model over time (Figure 4D–F). Figure 4 highlights a suite of time-dependent changes in the fecal metabolite relative abundances, especially those derived from the gut microbiota metabolism of the rice bran over a 4-week period in humans and in the colon cancer mouse model over a 14-week time period.

## 4. Discussion

This investigation demonstrated direct and indirect time-dependent relationships between host and gut microbiota metabolism following diet implementation of rice bran which protected against AOM/DSS-induced CRC in a murine model. The cecum and colonic tissue microbiota and metabolite changes after rice bran consumption were previously documented in conventional and germ-free mice [17]. The prior AOM/DSS-induced CRC study in conventional mice showed rice bran’s protective anti-cancer effect against inflammation (Table S1) [17], which was indicated by smaller-sized neoplastic lesions compared to the controls. Of importance also was an ~50% decrease in the presence of mucosal inflammatory cells, together with an increase in mucin-secreting goblet cells and more tight cellular junctions in the colons of mice consuming rice bran compared to the controls (Table S1) [17]. In the present study, fecal microbiota and metabolite changes in the control and rice bran groups were compared to adult CRC survivors. Similar to the results from the AOM/DSS murine model, human BENEFIT study participants had increased microbial richness and diversity after 2 and 4 weeks of rice bran consumption compared to the baseline and without changes in the control group [20]. 

CRC is closely correlated with diets rich in fiber from whole grains, and evidence supports gut microbial-mediated mechanisms against CRC progression [56,57]. The protective association between dietary fiber and CRC is influenced by the fermentation of fiber by bacteria in the colon and the formation of SCFAs [58]. High-fiber diets have demonstrated the ability to alter gut and fecal microbiota composition and promote bacterial metabolism for SCFA production [47,56,59], such as with the *Lachnospiraceae* family [60]. This study supports the enrichment of *Eubacterium xylanophilum group* and *Lachnospiraceae NK4B4 group* (both members of the *Lachnospiraceae* family) in the rice bran group mice. In a previous dietary wheat bran study, *Eubacterium xylanophilum group* was enriched for fermenting xylan, as well as associated with the production of metabolites such as formate, acetate, and butyrate [59,61]. Other bacteria of the *Lachnospiraceae* family were also enriched in the cecum and colon tissue microbiomes of these rice bran-fed mice [17]. The *Lachnospiraceae* family is abundant in the digestive tracts of mammals, and their butyrate production through dietary fiber fermentation has been associated with CRC protection [62]. Other studies also support associations between SCFA-producing bacteria related to rice bran consumption and CRC prevention [11,25,63]. Butyrate produced by gut microbes has previously been found to ameliorate the development of colitis by epigenetically regulating the transcription of genes responsible for regulatory T cells over a 4-week period in mice [64]. Butyrate has histone deacetylase inhibitor activity in colonocytes and promotes the transcription of tumor suppressor genes that all mechanistically work together to reduce inflammation and tumor growth [65]. Rice bran-mediated increases in beneficial bacteria for protection against CRC occurred without increased fecal SCFAs in humans. This is likely attributed to SCFA uptake directly by colonocytes, as reported by Oliver et al., where a high-fiber dietary intervention significantly altered the human gut microbiome after two weeks; however, it did not increase fecal SCFAs [66]. 

*Akkermansia* increased in the rice bran group mice in the first few weeks and has been associated with various positive health benefits. *Akkermansia* utilizes colonic mucin as its substrate to help regulate the intestinal mucus layer [67]. A high relative abundance of *Akkermansia* has been linked with a lower risk of obesity [68] and improved colon health [69], and studies have shown that higher levels of inflammation in the colon were associated with reduced abundances of *Akkermansia* [70]. It is also thought that *Akkermansia* may contribute to the production of SCFAs, such as propionate, and to the protection and integrity of epithelial cells [71]. Interestingly, the BENEFIT study evaluating CRC survivors following rice bran intake for 4 weeks found that rice bran supplementation increased levels of the SCFAs propionate and acetate after 14 days but not at 28 days [20]. It is estimated that 90% of SCFAs are absorbed in the colon or potentially used in cross-feeding with other microbiota, though their production is imperative in epithelial cell integrity and immune function regulation [72]. 

The time series of murine fecal metabolite changes during the inhibition of colitis-associated CRC were prominent between 6 and 14 weeks and had overlapping metabolic pathways of relevance to human fecal metabolite changes with rice bran consumption. Metabolites that decreased in the human feces and this murine model include N-acetylhistamine and ethylmalonate. N-acetylhistamine is a gut microbial-derived metabolite of histidine metabolism and can act as a stimulant of gastric secretion [73]. High levels of ethylmalonate observed in the rice bran group mice may indicate improved fatty acid transport associated with rice bran. Ethylmalonate results from the breakdown of butyrate and was associated with metabolic pathway alterations and some fatal forms of cancers in observational studies [74]. Carnitine is essential in transporting long-chain fatty acids into the mitochondria for beta oxidation; however, when carnitine levels are low, such as has been documented in a disease or cancerous state, long-chain fatty acids are processed outside of the mitochondria and thereby may be responsible for increased levels of ethylmalonate [75]. Notably, a microbial-derived metabolite from lignans increased in abundance for both people and mice consuming rice bran. Lignans consumed in the diet and from whole grains rely on gut microbiota, such as *Ruminococcus* spp., for conversion to active metabolites such as enterolactone [76,77]. Several other gut microbial species may also be predictors of enterolactone levels, which can enhance the breakdown of lignans via multiple reactions [78,79]. In the “Danish Diet, Cancer and Health” cohort study investigating Danish adults diagnosed with CRC, enterolactone was associated with a lower mortality risk in women, though not in men [80]. Other observational studies have shown strong associations between increased enterolactone levels and improved survival outcomes in men and women ranging from CRC, to lung, prostate, and breast cancers [81]. 

Figure 4B depicts statistically significant decreases in beta-hydroxyisovalerate in the rice bran group mice over time. High levels of beta-hydroxyisovalerate are a documented indicator of biotin deficiency and associated with a disease state [82]. In the rice bran treatment mice, there were decreased levels of beta-hydroxyisovalerate, which could indicate less biotin deficiency and be related to the gut microbiota production of biotin from the fermentation of rice bran. This was demonstrated by microbes such as *Bacteroides fragilis* and *Prevotella copri*. Other significant metabolite profile changes associated with rice bran-mediated inhibition of colon carcinogenesis included the TCA cycle intermediates malate, citrate, and aconitase. Rice bran is a rich source of malate and citrate [83] and may have contributed to these increases in the rice bran treatment group. Citrate is a promising metabolite for tumor growth suppression [84] via its ability to regulate immune function [85]. Cancer cells can bypass the TCA cycle and primarily utilize aerobic glycolysis. Thus, the increased TCA metabolites in the rice bran mouse feces over time suggest an altered intestinal microenvironment and the depletion of cancer cells. Arabinose was also notably changed following rice bran consumption and is largely found within plant polysaccharides and promotes the growth of *Bifidobacterium* and *Lactobacillus* alongside increased SCFA production [86]. Arabinose supplementation previously reduced DSS-induced colitis-associated inflammation and altered the diversity of gut microbiota related to inflammation [87] and remains a promising component from rice bran.

Fatty acids, such as medium-chain (e.g., carporate) and long-chain (e.g., trans-nonadecenoate (tr 19:1)) fatty acids, were lower in the rice bran mouse feces compared to the controls (Table 1). Interestingly, polyunsaturated fatty acids (PUFAs), linoleate (18:2n6), and linoleate alpha or gamma (18:3n3 or 6) were higher in the rice bran mice at weeks 10 and 14, suggesting PUFA accumulation from the rice bran. Similar to findings from the colon tissue of these mice [17], a significant increase in several dicarboxylate fatty acids (such as 2-hydroxyglutarate adipate (C6-DC), suberate (C8-DC), azelate (C9-DC), and octadecenedioate (C18:1-DC)) was noted in the feces at weeks 10 and 14.

An assortment of vitamins increased after rice bran intake in the mice and humans that have associations with antioxidant function and cancer prevention [47], including thiamin, nicotinate, pantothenate, vitamin B6 derivatives, vitamin E, and vitamin A [88,89,90,91,92,93]. In particular, pyridoxine (vitamin B6) increased 16.86-fold and 41.89-fold in the rice bran treatment, a vitamin which has coenzymatic functions that influence CRC carcinogenesis. A large case–control study reported the strong inverse and dose-dependent relationship between vitamin B6 intake and CRC risk [94] and was supported by other studies indicating anti-inflammatory properties for vitamin B6 [95,96]. Alpha- and gamma-tocotrienol (vitamin E isoforms) also increased in the rice bran treatment groups and are highlighted for their potent antioxidant capabilities and powerful anti-cancer properties [97]. Gamma-tocotrienol induced apoptosis in SW620 human colon carcinoma cells [98] and significantly decreased pro-inflammatory cytokines in a murine AOM/DSS CRC-induced model, thereby exhibiting anti-inflammatory properties [99]. Gamma-tocotrienol derived from rice bran was also cytotoxic to chemoresistant H28 cells in malignant mesothelioma [100]. Interestingly, the elevated levels of alpha-tocotrienol and gamma-tocotrienol in feces did not correlate with the levels in colonic tissue [17], highlighting the temporal and spatial differences for absorption of bioactive vitamins and the need for evaluating a spectrum of vitamins in food combinations.

Several phenolic compounds derived from rice bran have also been shown to possess cancer protection in animal and cell culture studies [13]. The accumulation of several phenolic compounds (including 2-hydroxyhippuarte, 3-(3-hydroxyphenyl)propionate, and 3-phenylpropionate) was observed in the rice bran group mice. Phenolics were more pronounced at the 14-week time point in relation to 10 weeks (vs. the control) and supported temporal differences in metabolism by microbiota. This was also seen in Figure 3E and the correlations between specific microbiota and metabolites. *Ruminococcaceae UCG 014,* from the *Ruminococcaceae* family and associated with fiber degradation, was also strongly correlated with metabolites such as salicylurate, enterolactone, and ferulic acid 4-sulfate, which are plant-derived [101]. Changes to benzoate metabolites in feces also reflect gut microbial metabolic changes following dietary rice bran intake and highlight the inter-individual differences in responses to rice bran intake in people related to the composition of intestinal microbiota and CRC risk [102]. Phenolics such as 3-phenylpropionate (hydrocinnamate), 2-oxindole-3-acetate, and ferulic acid 4-sulfate in murine feces were consistent with observations in the colon tissues of these mice; however, these phenolic metabolites were found in low abundances or not present at all in the colon tissues of germ-free AOM/DSS mice fed rice bran. This supports the gut microbiome’s necessity and relationship in producing various phenolic compound metabolites [17,103]. Ferulate, a phenolic compound associated with rice bran, has demonstrated antioxidant capabilities [104] and was increased in the feces of the rice bran treated mice. Ferulic acid derivates represent a suite of promising cancer-preventive agents by blocking the release of inflammatory TNF-α and a series of pathways related to anti-inflammatory immune responses. Decreasing colonic inflammation via rice bran-derived phenolics, fatty acids, vitamins, prebiotics, and TCA cycle intermediates is profound for global CRC protection via dietary measures. 

Study limitations for this murine model relate to the incomplete representation of CRC development in humans and that mice are cecal fermenters with limited differences across the entire colon. Other limitations include differences in environmental, dietary, and genetic factors in the human studies that influenced the gut microbiome compared to the murine model, which was a controlled environment. This study also included only male mice, and disease progression varies in female mice due to physiological and hormonal differences. Future studies should direct attention to dietary responders and non-responders to rice bran with respect to variations in lifestyle risk factors, body mass index, and microbiota composition, including bacterial drivers shown herein as cancer-protective. The fecal metabolites with similarities observed between adult males and females and in mice following rice bran intake supported the identification of diet-related cancer prevention biomarkers which may have future utility in population-based studies. 

## 5. Conclusions

This study reports a suite of fecal microbiome and metabolome changes in response to dietary rice bran intake during colon carcinogenesis, which support CRC reduction. Bacterial drivers of dietary rice bran-mediated effects included enriched *Eubacterium xylanophilum*, *Lachnospiraceae*, and *Ruminococcaceae*. Metabolite shifts included ethylmalonate, 2-hydroxyhippurate (salicylurate), enterolactone and ferulic acid 4-sulfate that were correlated with microbiota. Rice bran delivered vitamin B6 and vitamin E isomers with anti-cancer properties. Increased stool enterolactone was a microbial metabolite biomarker that increased in mice and humans consuming rice bran. These findings provide novel insight for integrated dietary and microbial metabolic mechanisms with serial changes over 14 weeks, whereby rice bran inhibited colon carcinogenesis. Furthermore, rice bran is an underutilized co-product of white rice processing that is nutrient-rich and affordable for widespread public health impacts to control CRC when compared to single nutrient supplementation strategies. Incorporating rice bran into daily meals provides a practical means for assessing CRC control and prevention in larger cohorts and for eventual dietary assessment in nutritional epidemiology and cancer prevention studies.

## Figures and Tables

**Table 1:** Fold differences in the median scaled relative abundances between the AOM/DSS rice bran group versus control mice at weeks 10 and 14. Metabolites with a fold difference cut-off of >1.5 or <0.5 are shown for either week 10, 14, or both.

Chemical Class	Metabolic Pathway	Metabolite	10 Weeks	14 Weeks
Amino Acids	Alanine and Aspartate Metabolism	N-acetylaspartate (NAA)	**1.62↑**	2.34↑*
	Glutamate Metabolism	Carboxyethyl-GABA	6.97↑*	10.85↑*
	Polyamine Metabolism	Agmatine	0.18↓*	0.16↓*
		Spermidine	0.38↓*	0.63↓
		Diacetylspermidine	0.37↓*	1.12↓
Carbohydrates	Pentose Metabolism	Arabinose	9.61↑*	34.58↑*
Energy	TCA Cycle	Malate	1.54↑*	1.74↑*
		Citrate	6.70↑*	20.56↑*
		Aconitate	2.54↑	10.72↑*
	Oxidative Phosphorylation	Phosphate	1.13↑	2.98↑*
Lipids	Fatty Acid Synthesis	Malonate	1.82↑*	2.59↑*
	Short-Chain Fatty Acid	Valerate (5:0)	**0.44↓**	0.29↓*
	Medium-Chain Fatty Acid	Caproate (6:0)	0.40↓*	0.46↓*
		Caprylate (8:0)	0.81↓	0.31↓*
	Long-Chain Fatty Acid	Trans-nonadecenoate (tr 19:1)	0.35↓*	0.34↓*
		Eicosenoate (20:1)	0.47↓*	0.39↓*
		Erucate (22:1n9)	0.49↓*	0.40↓*
	Polyunsaturated Fatty Acid (n3 and n6)	Linoleate (18:2n6)	1.45↑*	1.84↑*
		Linolenate (alpha or gamma; (18:3n3 or 6))	1.7↑*	2.22↑*
	Fatty Acid, Dicarboxylate	2-hydroxyglutarate	2.05↑*	3.65↑*
		Pimelate (C7-DC)	0.84↓	3.72↑*
		Suberate (C8-DC)	1.31↑*	1.69↑*
		Azelate (C9-DC)	1.48↑*	2.30↑*
		Hexadecanedioate (C16-DC)	1.75↑*	1.63↑*
		Octadecanedioate (C18-DC)	1.54↑*	1.76↑*
		Octadecenedioate (C18:1-DC)	11.24↑*	12.04↑*
		Octadecadienedioate (C18:2-DC)	0.77↓	0.38↓*
		Eicosanodioate (C20-DC)	1.55↑*	1.52↑*
	Fatty Acid Metabolism (Acyl Carnitine)	Palmitoylcarnitine (C16)	0.72↓	0.35↓*
		Eicosenoylcarnitine (C20:1)	0.75↓	0.41↓*
		Margaroylcarnitine (C17)	0.75↓	0.40↓*
		Stearoylcarnitine (C18)	0.84↓	0.46↓*
	Fatty Acid Monohydroxyl	16-hydroxypalmitate	7.09↑*	12.32↑*
		2-hydroxybehenate	**0.44↓**	0.46↓*
	Fatty Acid, Dihydroxy	9,10-DiHOME	1.15↑	1.61↑*
		Choline phosphate	**1.97↑**	2.49↑*
	Lysophospholipid	1-palmitoyl-GPI (16:0)	2.04↑	2.07↑*
		2-stearoyl-GPE (18:0)	0.38↓*	0.29↓*
	Glycerolipid Metabolism	Glycerol 3-phosphate	2.66↑*	2.64↑*
	Monoacylglycerol	1-oleoylglycerol (18:1)	2.70↑*	3.89↑*
		1-linoleoylglycerol (18:2)	2.83↑*	4.48↑*
		2-oleoylglycerol (18:1)	3.15↑*	5.12↑*
		2-linoleoylglycerol (18:2)	2.84↑*	4.53↑*
	Diacylglycerol	Oleoyl-linoleoyl-glycerol (18:1/18:2) [2]	6.18↑*	2.55↑*
	Sphingolipid Synthesis	Sphingadienine	0.96↓	0.45↓*
	Dihydroceramides	N-stearoyl-spinganine (d18:0/18:0)	0.79↓	0.45↓*
	Ceramides	N-stearoyl-sphingosine (d18:1/18:0)	**0.68↓**	0.49↓*
	Mevalonate Metabolism	3-hydroxy-3-mehtylglutarate	1.38↑	3.00↑*
	Sterol	Lanosterol	9.92↑*	4.95↑*
		4-cholesten-3-one	1.98↑*	1.73↑*
	Primary Bile Acid Metabolism	Glycocholate sulfate	0.19↓*	0.22↓*
	Secondary Bile Acid Metabolism	Taurochenodeoxycholate sulfate	**0.12↓**	0.05↓*
		Isohyodeoxycholate	1.47↑*	1.92↑*
Cofactors and Vitamins	Nicotinate and Nicotinamide Metabolism	Quinolinate	1.93↑*	3.92↑*
		Nicotinate ribonucleoside	3.30↑*	10.24↑*
		1-methylnicotinamide	0.93↓	4.03↑*
		Trigonelline (N’-methylnicotinate)	2.74↑*	4.00↑*
	Pantothenate and CoA Metabolism	Pantetheine	2.53↑*	2.50↑*
	Tocopherol Metabolism	Delta-tocopherol	0.50*	0.37↓*
		Alpha-tocotrienol	1.70↑*	1.59↑*
		Gamma-tocotrienol	1.64↑*	1.46↑*
		Gamma-tocopherol/Beta-tocopherol	0.44↓*	0.33↓*
	Hemoglobin and Porphyrin Metabolism	Protoporphyrin IX	1.74↑*	1.74↑*
		Bilirubin (Z,Z)	0.23↓*	0.20↓*
		Bilirubin (E,E)*	0.18↓*	0.20↓*
		Biliverdin	0.34↓*	0.36↓*
	Thiamin Metabolism	Thiamin (Vitamin B1)	1.29↑	1.77↑*
	Vitamin A Metabolism	Retinol (Vitamin A)	1.25↑	2.42↑*
		Carotene diol (1)	3.76↑*	3.04↑*
	Vitamin B6 Metabolism	Pyridoxine (Vitamin B6)	16.86↑*	41.89↑*
		Pyridoxate	**1.40↑**	2.07↑*
		Pyridoxamine	1.60↑*	1.67↑*
Xenobiotics	Benzoate Metabolism	2-hydroxyhippurate (salicylurate)	11.69↑*	65.75↑*
		Phenylpropionylglycine	3.14↑	10.2↑*
		3-(3-hydroxyphenyl)propionate sulfate	1.30↑	38.24↑*
		3-(3-hydroxyphenyl)propionate	7.03↑*	19.12↑*
		3-phenylpropionate (hydrocinnamate)	6.31↑*	12.38↑*
	Food Component/Plant	Cinnamoylglycine	3.24↑	13.21↑*
		2-oxindole-3-acetate	7.51↑*	7.51↑*
		Dihydroferulic acid	3.42↑*	6.66↑*
		Enterolactone	3.37↑*	3.27↑*
		Ferulate	7.38↑*	27.13↑*
		Ferulic Acid 4-sulfate	0.69↓	24.21↑*
		Pheophytin A	38.38↑*	37.25↑*
		Pinosylvin	3.46↑*	3.36↑*
		Sinapate	3.75↑*	4.99↑*
		4-hydroxycinnamate	**2.01↑**	4.66↑*
	Drug–Tropical Agent	Salicylate	2.13↑*	6.23↑*

* Table values indicate metabolites with a statistically different (*p* < 0.05) relative abundance between the rice bran and control treatments at 10 or 14 weeks. **Bold** table values indicate metabolites with q < 0.15. ↑ indicates that a metabolite abundance was higher in the rice bran treatment groups as compared to the control and ↓ indicates metabolite abundance was lower in the rice bran treatment group as compared to the control.

**Table 2:** Human and murine fecal metabolite changes following dietary rice bran intake. Beans/Bran Enriching Nutritional Eating for Intestinal Health Trial (BENEFIT) rice bran group at 4 weeks compared to the baseline are shown alongside AOM/DSS mice fed rice bran with changes at 2, 6, 10, and 14 weeks compared to the baseline.

Metabolic Pathway	Metabolite	BENEFIT Fold Change (4 Weeks/Baseline)	Mice Fold Change (2 Weeks/Baseline)	Mice Fold Change (6 Weeks/2 Weeks)	Mice Fold Change (10 Weeks/6 Weeks)	Mice Fold Change (14 Weeks/10 Weeks)
Histidine Metabolism	N-acetylhistamine	0.52↓	0. 13↓	0.18↓	0.98↓#	2.48↑
Leucine, Isoleucine, and Valine Metabolism	Beta-hydroxyisovalerate	17.9↑	0.28↓	0.60↓	0.98↓#	1.55↑
	Ethylmalonate	0.70↓	0.20↓	0.73↓#	1.12↓#	2.46↑
Methionine, Cysteine, SAM, and Taurine Metabolism	N-acetylmethionine sulfoxide	0.47↓	2.73↑	0.72↓#	1.15↑#	1.61↑
Gamma-glutamyl Amino Acid	Gamma-glutamylphenylalanine	1.02↑	0.29↓	0.76↓#	0.65↓#	2.36↑
Benzoate Metabolism	p-cresol sulfate	0.69↓	70.2↑	1.48↑	0.60↑#	1.69↑#
Food Component/Plant	Apigenin	15.32↑	3.15↑	3.86↑	0.43↓#	0.01↓
	Enterolactone	3.55↑	7.77↑	1.70↑	0.79↑#	0.90↓#

Note: ↑ designates metabolites with significantly (*p* ≤ 0.05) higher abundances of stool metabolomes at the end of the trial when compared with comparators as noted (metabolite ratio of ≥1.00). ↓ designates metabolites with significant (*p* ≤ 0.05) lower abundances in stool metabolomes at the end of the trial when compared with comparators as noted (metabolite ratio of <1.00). # = not statistically significant (*p* > 0.05).

## Data Availability

Sequence data supporting this manuscript are available under NCBI SRA BioProject Accession No. PRJNA516457. All data will be made available upon request to the corresponding author.

## Supplementary Materials

The following supporting information can be downloaded at: https://www.mdpi.com/article/10.3390/cancers15082231/s1. Figure S1. Non-parametric Kruskal-Wallis pairwise test of Observed OTUs between AOM DSS mice rice bran and control groups over time. Table S1. Rice bran meditated protective efficacy against AOM-DSS induced CRC. Table S2. (a) Alpha Diversity Kruskal-Wallis comparisons of all time points rice bran to control and (b) Beta Diversity PERMANOVA comparisons of all time points rice bran to control AOM DSS mice. Table S3. (a) Alpha diversity measures and (b) Beta diversity measures at Baseline, mice rice bran group compared to control AOM DSS mice. Table S4. Beta Diversity Unweighted UniFrac PERMANOVA Pairwise comparison, mice rice bran group compared to control at each time point. *P*-values in bold are <0.05. Table S5. Non-parametric Kruskal-Wallis observed OTUs between particular time points in mice. Bolded values indicate significance (*p* < 0.05). Table S6. Metabolites with significant fold difference in AOM/DSS mice rice bran group compared with control at week 10. Table S7. Metabolites with significant fold difference in AOM/DSS mice rice bran group compared with control at week 14. Table S8. Pearson Correlation Selected Microbes and Metabolites. Table S9. *P*-values and q-values for changes in metabolites in human Beans/Bran Enriching Nutritional Eating For Intestinal health Trial (BENEFIT) study rice bran group at 4 weeks compared to baseline and in AOM/DSS mice in the rice bran group at 6, 10, and 14 weeks and baseline. Metabolites listed in the table had a significant fold change in the BENEFIT trial (*p* < 0.05), and a significant fold change in at least 2 time points in the AOM/DSS mice and were not significantly different from control at baseline (*p* < 0.05).
