# Supplementary material for: Integrated Microbiota and Metabolite Changes following Rice Bran Intake during Murine Inflammatory Colitis-Associated Colon Cancer and in Colorectal Cancer Survivors
Source: Cancers (Basel). 2023 Apr 10;15(8):2231. doi: 10.3390/cancers15082231 (PMC10136752; doi:10.3390/cancers15082231)
Supplement: Supplementary file 1 [file cancers-15-02231-s001.zip › Revision2_Supplemental Files Cancers_27MAR2023.pdf]

Supplementary Files

Table S1. Rice bran meditated protective efficacy against AOM-DSS induced CRC

S. No.	Histopathology and molecular analysis of cancer-associated markers in colonic tissue	Outcomes (Rice bran diet vs. AOM-DSS controls)
1.	Tumor lesions	↓ Size of lesions
2.	Dysplasia	↓ High-grade dysplasia
3.	Epithelial layer	↓ Epithelial erosion
4.	Inflammatory infiltrates	↓ Sub-mucosal inflammation
5.	Goblet cells	↑ Presence of dense mucin rich goblet cells
6.	Ki-67 (% positive cells)	↓ Proliferation, $p \leq 0.001$
7.	β-catenin	↓ expression, $p \leq 0.05$
8.	CD44	↓ expression, $p \leq 0.05$
9.	ZO-1	↑ expression, $p \leq 0.01$
10.	Claudin-2	↓ expression, $p \leq 0.01$
11.	Cox-2	↓ expression, $p \leq 0.001$
12.	NF-κB (p65)	↓ expression, $p \leq 0.01$

Adapted from *Kumar et al., Mol Carcinog. 2022 Oct;61(10):941-957. doi: 10.1002/mc.23452. PMID: 35856887*

Table S2. (a) Alpha Diversity Kruskal-Wallis comparisons of all time points rice bran to control and (b) Beta Diversity PERMANOVA comparisons of all time points rice bran to control AOM DSS mice.

a)

**Alpha Diversity Measure**	P-value
Evenness	0.685
Faith PD	0.055
Observed OTUs	0.025*
Shannon	0.303

b)

**Beta Diversity Measure**	P-value	Test statistic
Bray Curtis	0.002*	3.84
Weighted Unifrac Distance Matrix	0.004*	3.78
Jaccard Distance	0.001*	3.39
Unweighted Unifrac Distance Matrix	0.008*	3.13

Table S3. (a) Alpha diversity measures and (b) Beta diversity measures at Baseline, mice rice bran group compared to control AOM DSS mice.

a)

**Alpha Diversity Measure**	Kruskal-Wallis Pairwise comparison
Evenness	p=1.00
Faith PD	p=1.00
Observed OTUs	p=0.773
Shannon	p=0.773

b)

**Beta Diversity Measure**	PERMANOVA Pairwise comparison
Bray Curtis	p=0.031*
Weighted Unifrac Distance Matrix	p=0.035*
Jaccard Distance	p=0.260
Unweighted Unifrac Distance Matrix	p=0.308

* Indicates significant difference (P<0.05)

Table S4. Beta Diversity Unweighted UniFrac PERMANOVA Pairwise comparison, mice rice bran group compared to control at each time point. P-values in bold are <0.05.

Week	p-value	q-value
Baseline	0.308	0.3465
2	**0.031**	0.0462
6	**0.029**	0.0462
10	**0.032**	0.0462
14	**0.039**	0.0462

Table S5. Non-parametric Kruskal-Wallis observed OTUs between particular time points in mice. Bolded values indicate significance ($p<0.05$).

Group 1	Group 2	p-value	q-value
Rice bran Baseline	Rice bran week 2	**0.02**	0.04
	Rice bran week 6	**0.02**	0.04
	Rice bran week 10	**0.04**	0.08
	Rice bran week 14	**0.02**	0.04
	Control Baseline	0.77	0.79
	Control week 2	**0.02**	0.04
	Control week 6	**0.02**	0.04
	Control week 10	**0.02**	0.04
	Control week 14	**0.02**	0.04
Rice bran week 14	Rice bran week 2	**0.04**	0.08
	Rice bran week 6	0.19	0.32
	Rice bran week 10	0.77	0.79

Figure S1. Non-parametric Kruskal-Wallis pairwise test of Observed OTUs between AOM DSS mice rice bran and control groups over time.

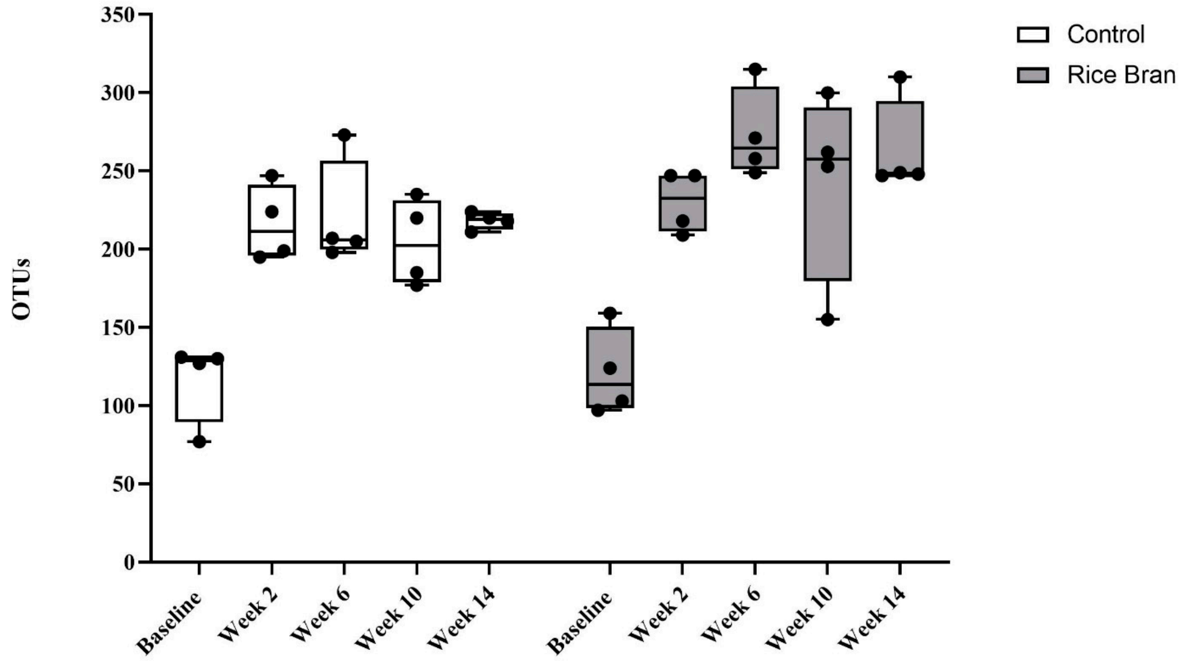

Table S6. Metabolites with significant fold difference in AOM/DSS mice rice bran group compared with control at week 10.

Chemical Class	Metabolic Pathway	Metabolite	Fold Difference (Rice Bran 10 Weeks/Control 10 Weeks)	*p*-value	*q*-value
Amino Acid	Glycine, Serine and Threonine Metabolism	Glycine	0.61↓	0.0025	0.0103
		Serine	0.70↓	0.0042	0.0151
		Threonine	0.70↓	0.0098	0.0274
	Alanine and Aspartate Metabolism	Alanine	0.71↓	0.0043	0.0151
	Glutamate Metabolism	carboxyethyl-GABA	6.97↑	0.0000	0.0000
		N-methyl-GABA	0.18↓	0.0007	0.0039
		S-1-pyrroline-5-carboxylate	0.67↓	0.0084	0.0247
	Histidine Metabolism	N-acetyl-3-methylhistidine*	0.44↓	0.0129	0.0336
		trans-urocanate	0.64↓	0.0016	0.0078
		Formiminoglutamate	0.65↓	0.0398	0.0796
	Lysine Metabolism	N,N,N-trimethyl-5-aminovalerate	1.5↑	0.0392	0.0794
	Phenylalanine Metabolism	Phenylalanine	0.67↓	0.0001	0.0005
	Tyrosine Metabolism	Tyrosine	0.68↓	0.0037	0.0134
	Tryptophan Metabolism	Tryptophan	0.61↓	0.005	0.0027
		Picolinate	0.58↓	0.0022	0.0094
	Leucine, Isoleucine and Valine Metabolism	Leucine	0.63↓	0.0000	0.0002
		Isoleucine	0.60↓	0.0000	0.0000
		Valine	0.63↓	0.0002	0.0012
	Methionine, Cysteine, SAM and Taurine Metabolism	Methionine	0.73↓	0.0065	0.0209
		N-formylmethionine	0.48↓	0.0421	0.0808
		cysteine s-sulfate	0.38↓	0.0002	0.0014
		cysteine sulfinic acid	0.48↓	0.0021	0.0093
	Urea cycle; Arginine and Proline Metabolism	Arginine	0.63↓	0.0064	0.0209
		Ornithine	0.51↓	0.0355	0.0734
		Proline	0.65↓	0.0149	0.0382
		N-acetylproline	0.54↓	0.0051	0.0177
		N-methylproline	2.23↑	0.0000	0.0005
	Polyamine Metabolism	Agmatine	0.18↓	0.0000	0.0002
		Spermidine	0.38↓	0.0067	0.0211
		diacetylspermidine*	0.37↓	0.0247	0.0551
	Glutathione Metabolism	cysteinylglycine	0.29↓	0.0067	0.0211
Peptide		gamma-glutamylhistidine	1.88↑	0.0125	0.0329

	Gamma-glutamyl Amino Acid	gamma-glutamylphenylalanine	0.55↓	0.0419	0.0808
	Dipeptide	glycylisoleucine	0.71↓	0.0096	0.0270
		glycylleucine	0.70↓	0.0006	0.0032
		histidylalanine	0.55↓	0.0004	0.0026
		isoleucylglycine	0.75↓	0.0070	0.0215
		leucylalanine	0.69↓	0.0418	0.0808
		phenylalanylalanine	0.52↓	0.0001	0.0009
		prolylglycine	0.47↓	0.0064	0.0209
		tryptophylglycine	0.66↓	0.0437	0.0813
		tyrosylglycine	0.59↓	0.0001	0.0005
		valylglutamine	0.72↓	0.0032	0.0124
		leucylglutamine*	0.76↓	0.0081	0.0241
Carbohydrate	Pentose Metabolism	Arabinose	9.61↑	0.0000	0.0001
	Glycogen Metabolism	Maltose	0.39↓	0.0346	0.0719
	Fructose, Mannose, and galactose Metabolism	Fructose	0.50↓	0.0023	0.0099
Energy	TCA Cycle	Malate	1.54↑	0.0154	0.0391
		Citrate	6.70↑	0.0199	0.0473
Lipid	Fatty Acid Synthesis	Malonate	1.82↓	0.0031	0.0121
	Medium Chain Fatty Acid	caproate (6:0)	0.40↓	0.0252	0.0554
	Long Chain Fatty Acid	palmitoleate (16:1n7)	0.78↓	0.0434	0.0813
		10-nonadecenoate (19:1n9)	0.62↓	0.0483	0.0856
		trans-nonadecenoate (tr 19:1)*	0.35↓	0.0002	0.0012
		eicosenoate (20:1)	0.47↓	0.0251	0.0554
		erucate (22:1n9)	0.49↓	0.0185	0.0451
	Polyunsaturated Fatty Acid (n3 and n6)	linoleate (18:2n6)	1.45↑	0.0213	0.0501
		linolenate [alpha or gamma; (18:3n3 or 6)]	1.7↑	0.0009	0.0047
		arachidonate (20:4n6)	0.55↓	0.0061	0.0208
		docosadienoate (22:2n6)	0.59↓	0.0476	0.0856
	Fatty Acid, Dicarboxylate	2-hydroxyglutarate	2.05↑	0.0180	0.0442
		suberate (C8-DC)	1.31↑	0.0480	0.0856
		azelate (C9-DC)	1.48↑	0.0121	0.0320
		undecanedioate (C11-DC)	0.36↓	0.0309	0.0650
		tetradecanedioate (C14-DC)	0.44↓	0.0000	0.0000
		hexadecanedioate (C16-DC)	1.75↑	0.0024	0.0100
		octadecanedioate (C18-DC)	1.54↑	0.0197	0.0473

	octadecenedioate (C18:1-DC)*	11.24↑	0.0000	0.0000
	eicosanodioate (C20-DC)	1.55↑	0.00785	0.0227
Fatty Acid Metabolism(Acyl Carnitine)	acetylcarnitine (C2)	0.27↓	0.0020	0.0092
Carnitine Metabolism	deoxycarnitine	0.51↓	0.0003	0.0016
Fatty Acid Monohydroxyl	16-hydroxypalmitate	7.09↑	0.0000	0.0000
	hydroxystearate sulfate	0.54↓	0.0113	0.0304
Fatty Acid, Dihydroxy	12,13-DiHOME	1.89↑	0.0236	0.0533
Phospholipid Metabolism	glycerophosphoethanolamine	0.61↓	0.0017	0.0080
Phosphatidylcholine	1-oleoyl-2-linoleoyl-GPC (18:1/18:2)*	3.73↑	0.0001	0.0010
Phosphatidylethanolamine (PE)	1-palmitoyl-2-oleoyl-GPE (16:0/18:1)	2.35↑	0.0024	0.0100
Lysophospholipid	1-palmitoyl-GPI (16:0)	2.04↑	0.0065	0.0209
Plasmalogen	1-(1-enyl-palmitoyl)-2-palmitoyl-GPC (P-16:0/16:0)*	0.59↓	0.0491	0.0863
Lysoplasmalogen	1-(1-enyl-palmitoyl)-GPC (P-16:0)*	0.62↓	0.0481	0.0856
	1-(1-enyl-palmitoyl)-GPE (P-16:0)*	0.50↓	0.0032	0.0124
	1-(1-enyl-stearoyl)-GPE (P-18:0)*	0.52↓	0.0075	0.0227
Glycerolipid Metabolism	Glycerol	1.43↑	0.0418	0.0808
	glycerol 3-phosphate	2.66↑	0.0016	0.0078
Monoacylglycerol	1-oleoylglycerol (18:1)	2.7↑	0.0002	0.0011
	1-oleoylglycerol (18:1)	2.83↑	0.0001	0.0007
	2-oleoylglycerol (18:1)	3.15↑	0.0001	0.0008
	2-linoleoylglycerol (18:2)	2.84↑	0.0021	0.0093
Diacylglycerol	palmitoyl-linoleoyl-glycerol (16:0/18:2) [2]*	4.21↑	0.0031	0.0123
	oleoyl-linoleoyl-glycerol (18:1/18:2) [2]	6.18↑	0.0002	0.0012
Ceramides	N-palmitoyl-sphingosine (d18:1/16:0)	0.63↓	0.0020	0.0092
	N-(2-hydroxypalmitoyl)-sphingosine (d18:1/16:0(2OH))	0.59↓	0.0024	0.0101

		ceramide (d18:1/14:0, d16:1/16:0)*	0.63↓	0.0270	0.0590
		ceramide (d18:1/17:0, d17:1/18:0)*	0.55↓	0.0068	0.0212
		ceramide (d18:1/20:0, d16:1/22:0, d20:1/18:0)*	0.69↓	0.0278	0.0599
		ceramide (d18:2/24:1, d18:1/24:2)*	0.50↓	0.0438	0.0813
	Dihydrosphingomyelins	palmitoyl dihydrosphingomyelin (d18:0/16:0)*	0.62↓	0.0095	0.0270
	Sphingomyelins	palmitoyl sphingomyelin (d18:1/16:0)	0.55↓	0.0013	0.0068
		sphingomyelin (d17:1/16:0, d18:1/15:0, d16:1/17:0)*	0.47↓	0.0288	0.0616
	Sterol	lanosterol	9.92↑	0.0000	0.0000
		4-cholesten-3-one	1.98↑	0.0060	0.0206
		beta-sitosterol	0.72↓	0.0159	0.0393
		7-hydroxycholesterol (alpha or beta)	0.46↓	0.0444	0.0820
	Primary Bile Acid Metabolism	glycocholate sulfate	0.19↓	0.0034	0.0130
	Secondary Bile Acid Metabolism	hyocholate	0.62↓	0.0411	0.0808
		isohyodeoxycholate	1.47↑	0.0455	0.0834
Nucleotide	Pyrimidine Metabolism, Cytidine containing	cytidine 2',3'-cyclic monophosphate	0.10↓	0.0430	0.0813
Cofactors and Vitamins	Nicotinate and Nicotinamide Metabolism	quinolinate	1.93↑	0.0089	0.0258
		nicotinate ribonucleoside	3.30↑	0.0484	0.0856
		trigonelline (N'-methylnicotinate)	2.74↑	0.0148	0.0382
	Pantothenate and CoA Metabolism	pantethine	2.53↑	0.0009	0.0047
		pantetheine	1.83↑	0.0211	0.0500
	Ascorbate and Aldarate Metabolism	oxalate (ethanedioate)	2.37↑	0.0000	0.0000
		dehydroabietic acid	1.84↑	0.0370	0.0754
	Tocopherol Metabolism	delta-tocopherol	0.50↓	0.0001	0.0007
		alpha-tocotrienol	1.7↑	0.0001	0.0005
		gamma-tocotrienol	1.64↑	0.0039	0.0142
		gamma-tocopherol/beta-tocopherol	0.44↓	0.0000	0.0000
		protoporphyrin IX	1.74↑	0.0277	0.0599

		bilirubin (Z,Z)	0.23↓	0.0002	0.0015
	Hemoglobin and Porphyrin Metabolism	bilirubin (E,E)*	0.18↓	0.0000	0.0004
		biliverdin	0.34↓	0.0102	0.0279
		L-urobilin	2.1↑	0.0036	0.0134
	Vitamin A Metabolism	carotene diol (1)	3.76↑	0.0000	0.0000
	Vitamin B6 Metabolism	pyridoxine (Vitamin B6)	16.86↑	0.0000	0.0000
		pyridoxamine	1.6↑	0.0160	0.0401
Xenobiotics	Benzoate Metabolism	2-hydroxyhippurate (salicylurate)	11.69↑	0.0245	0.0551
		3-(3-hydroxyphenyl)propionate	7.03↑	0.0000	0.0000
		3-phenylpropionate (hydrocinnamate)	6.31↑	0.0000	0.0000
	Xanthine Metabolism	1-methylurate	0.43↓	0.0011	0.0055
	Food component/Plant	2-isopropylmalate	1.69↑	0.0308	0.0650
		2-oxindole-3-acetate	7.51↑	0.0000	0.0000
		dihydroferulic acid	3.42↑	0.0004	0.0024
		enterolactone	3.37↑	0.0001	0.0008
		Ferulate	7.38↑	0.0000	0.0000
		pheophytin A	38.38↑	0.0000	0.0000
		Pinosylvin	3.46↑	0.0012	0.0063
		Quinate	1.7↑	0.0360	0.0739
		Sinapate	3.75↑	0.0000	0.0001
		enterodiol	1.94↑	0.0466	0.849
	Drug – Tropical Agent	Salicylate	2.13↑	0.0016	00079
	Chemical	O-sulfo-L-tyrosine	0.29↓	0.0235	0.0533
		succinimide	0.65↓	0.0195	0.0471
		thioproline	0.54↓	0.0084	0.0247

Table S7. Metabolites with significant fold difference in AOM/DSS mice rice bran group compared with control at week 14.

Chemical Class	Metabolic Pathway	Metabolite	Fold Difference (Rice Bran 14 Weeks/Control 14 Weeks)	*p*-value	*q*-value
Amino Acid	Glycine, Serine and Threonine Metabolism	Glycine	0.62↓	0.0032	0.0240
		Serine	0.70↓	0.0033	0.0240
		Threonine	0.76↓	0.0416	0.1550
	Alanine and Aspartate Metabolism	Alanine	0.77↓	0.0260	0.1061
		N-acetylaspartate (NAA)	2.34↑	0.0033	0.0240
	Glutamate Metabolism	carboxyethyl-GABA	10.85↑	0.0000	0.0000
		N-methyl-GABA	0.43↓	0.0179	0.0821
	Histidine Metabolism	trans-urocanate	0.73↓	0.0211	0.0907
		Formiminoglutamate	0.61↓	0.0175	0.0814
	Lysine Metabolism	N-acetyl-cadaverine	0.54↓	0.0068	0.0388
		N,N,N-trimethyl-5-aminovalerate	1.80↑	0.0096	0.0518
	Phenylalanine Metabolism	Phenylalanine	0.83↓	0.0419	0.1503
	Tyrosine Metabolism	Gentisate	3.99↑	0.0009	0.0095
	Tryptophan Metabolism	Tryptophan	0.75↓	0.0397	0.1455
		Picolinate	0.56↓	0.0006	0.0069
	Leucine, Isoleucine and Valine Metabolism	Leucine	0.78↓	0.0091	0.0501
		Isovalerate (i5:0)	0.24↓	0.0044	0.9326
		Isoleucine	0.72↓	0.0008	0.0083
		Ethylmalonate	3.49↑	0.0267	0.1082
		Mathylsuccinate	2.42↑	0.0160	0.0763
		Valine	0.72↓	0.0050	0.0314
	Methionine, Cysteine, SAM and Taurine Metabolism	cysteine s-sulfate	0.52↓	0.0050	0.0314
		cysteine sulfinic acid	0.61↓	0.0046	0.0295
	Urea cycle; Arginine and Proline Metabolism	Arginine	0.74↓	0.0446	0.1503
		Proline	0.68↓	0.0192	0.0861
		N-acetylproline	0.71↓	0.0202	0.0885
		N-methylproline	2.22↑	0.0002	0.0025
	Polyamine Metabolism	Agmatine	0.16↓	0.0000	0.0001
		Spermidine	0.38↓	0.0067	0.0211
		diacetylspermidine*	0.37↓	0.0247	0.0551

	Glutathione Metabolism	cysteinylglycine	0.65↓	0.0431	0.1503
Peptide	Dipeptide	phenylalanylalanine	0.66↓	0.0063	0.0369
		prolylglycine	0.43↓	0.0017	0.0151
		valylglutamine	0.72↓	0.0032	0.0124
		leucylglutamine*	0.77↓	0.0081	0.0451
Carbohydrate	Pentose Metabolism	Arabinose	34.58↑	0.0000	0.0000
	Disaccharides and Oligosaccharides	Lactose	0.67↓	0.0440	0.1503
		Lactobionate	2.99↑	0.0066	0.0382
	Advanced Glycation End-product	N6-carboxymethyllysine	1.63↑	0.0434	0.1503
Energy	TCA Cycle	Malate	1.74↑	0.0042	0.0285
		Citrate	20.56↑	0.0003	0.0041
		Aconitate	10.72↑	0.0054	0.0335
	Oxidative Phosphorylation	Phosphate	2.98↑	0.0196	0.0899
Lipid	Fatty Acid Synthesis	Malonate	2.59↑	0.0000	0.0003
	Short Chain Fatty Acid	Valerate (5:0)	0.29↓	0.0099	0.0527
	Medium Chain Fatty Acid	Caproate (6:0)	0.46↓	0.0162	0.0169
		Heptanoate (7:0)	0.46↓	0.0488	0.1627
		Caprylate (8:0)	0.31↓	0.0212	0.0907
	Long Chain Fatty Acid	Palmitoleate (16:1n7)	0.70↓	0.0045	0.0294
		10-nonadecenoate (19:1n9)	0.53↓	0.0029	0.0217
		trans-nonadecenoate (tr 19:1)*	0.34↓	0.0001	0.0009
		eicosenoate (20:1)	0.39↓	0.0042	0.0285
		erucate (22:1n9)	0.40↓	0.0018	0.0151
	Polyunsaturated Fatty Acid (n3 and n6)	Docosahexaenoate (DHA, 22:6n3)	0.68↓	0.0498	0.1654
		linoleate (18:2n6)	1.84↑	0.0025	0.0197
		linolenate [alpha or gamma; (18:3n3 or 6)]	2.22↑	0.0000	0.0000
		arachidonate (20:4n6)	0.61↓	0.0097	0.0518
		Docosapentaenoate (n6 DPA; 22:5n6)	0.54↓	0.0322	0.1234
		docosadienoate (22:2n6)	0.56↓	0.0146	0.0722
	Fatty Acid, Branched	13-methylmyristate (i15:0)	0.54↓	0.0037	0.0261
		17-methylstearate (i19:0)	0.60↓	0.0096	0.0518
	Fatty Acid, Dicarboxylate	2-hydroxyglutarate	3.65↑	0.0003	0.0039
		Glutarate (C5-DC)	1.74↑	0.0358	0.1341
		Adipate (C6-DC)	2.49↑	0.0394	0.1455
		Pimelate (C7-DC)	3.72↑	0.0006	0.0070

	Suberate (C8-DC)	1.69↑	0.0070	0.0394
	Azelate (C9-DC)	2.30↑	0.0009	0.0091
	Tetradecanedioate (C14-DC)	0.54↓	0.0001	0.0010
	Hexadecanedioate (C16-DC)	1.63↑	0.0149	0.0924
	Dodecenedioate (C12:1-DC)	2.62↑	0.0320	0.1234
	Octadecanedioate (C18-DC)	1.76↑	0.0046	0.0295
	Octadecenedioate (C18:1-DC)*	12.04↑	0.0000	0.0000
	Octadecadienedioate (C18:2-DC)	0.38↓	0.0014	0.0126
	Eicosanodioate (C20-DC)	1.52↑	0.0138	0.0692
Fatty Acid Metabolism(Acyl Carnitine)	Palmitoylcarnitine (C16)	0.35↓	0.0002	0.0034
	Eicosenoylcarnitine (C20:1)	0.41↓	0.0010	0.0101
	Margaroylcarnitine (C17)	0.40↓	0.0003	0.0037
	Stearoylcarnitine (C18)	0.46↓	0.0005	0.0056
Carnitine Metabolism	deoxycarnitine	0.57↓	0.0024	0.0195
Fatty Acid Monohydroxyl	16-hydroxypalmitate	12.32↑	0.0000	0.0000
	2-hydroxybehenate	0.46↓	0.0165	0.0770
	3-hydroxyplamitate	0.62↓	0.0356	0.1341
	13-HODE + 9-HODE	1.9↑	0.0245	0.1011
Fatty Acid, Dihydroxy	9,10-DiHOME	1.61↑	0.0192	0.0861
	Choline phosphate	2.49↑	0.0125	0.0643
Phospholipid Metabolism	glycerophosphoethanolamine	0.59↓	0.0006	0.0069
Phosphatidylcholine	1,2-dipalmitoyl-GPC (16:0/16:0)	0.65↓	0.0275	0.1107
Phosphatidylethanolamine (PE)	1,2-dilinoleoyl-GPC (18:2/18:2)	1.65↑	0.0303	0.1188
Lysophospholipid	1-palmitoyl-GPI (16:0)	2.07↑	0.0060	0.0358
	1-palmitoyl-GPE (16:0)	0.53↓	0.0060	0.0358
	1-stearoyl-GPE (18:0)	0.64↓	0.0443	0.1503
	2-stearoyl-GPE (18:0)	0.29↓	0.0013	0.0118
Lysoplasmalogen	1-(1-enyl-palmitoyl)-GPC (P-16:0)*	0.62↓	0.0446	0.1503
	1-(1-enyl-palmitoyl)-GPE (P-16:0)*	0.47↓	0.0011	0.0103
	1-(1-enyl-stearoyl)-GPE (P-18:0)*	0.57↓	0.01298	0.0656
	1-(1-enyl-oleoyl)-GPE (P-18:1)*	0.58↓	0.0203	0.0885
Glycerolipid Metabolism	glycerol 3-phosphate	2.64↑	0.0025	0.0198

Monoacylglycerol	1-oleoylglycerol (18:1)	3.89↑	0.0000	0.0000
	1-linoleoylglycerol (18:2)	4.48↑	0.0000	0.0000
	2-oleoylglycerol (18:1)	5.12↑	0.0000	0.0001
	2-linoleoylglycerol (18:2)	4.53↑	0.0001	0.0016
	oleoyl-linoleoyl-glycerol (18:1/18:2) [2]	2.55↑	0.0041	0.0285
Sphingolipid Synthesis	Sphingadienine	0.45↓	0.0055	0.0340
	Hexadecasphinganine (d16:0)	0.53↓	0.0002	0.0032
Dihydroceramides	N-stearoyl-spinganine (d18:0/18:0)	0.45↓	0.0000	0.0006
Ceramides	N-palmitoyl-sphingosine (d18:1/16:0)	0.65↓	0.0026	0.0199
	N-(2-hydroxypalmitoyl)-sphingosine (d18:1/16:0(2OH))	0.65↓	0.0195	0.0866
	N-stearoyl-sphingosine (d18:1/18:0)	0.49↓	0.0028	0.0213
	ceramide (d18:1/14:0, d16:1/16:0)*	0.56↓	0.0069	0.0389
	ceramide (d18:1/17:0, d17:1/18:0)*	0.52↓	0.0019	0.0163
	ceramide (d18:1/20:0, d16:1/22:0, d20:1/18:0)*	0.61↓	0.0065	0.0377
Dihydrosphingomyelins	palmitoyl dihydrosphingomyelin (d18:0/16:0)*	0.64↓	0.0119	0.0619
Sphingomyelins	palmitoyl sphingomyelin (d18:1/16:0)	0.56↓	0.0011	0.0103
Sphingosines	Sphingosine	0.65↓	0.0288	0.1145
	Hexadecasphinganine (d16:0)	0.53↓	0.0002	0.0032
Mevalonate Metabolism	3-hydroxy-3-mehtylglutarate	3.00↑	0.0034	0.0243
Sterol	lanosterol	4.95↑	0.0000	0.0000
	4-cholesten-3-one	1.73↑	0.0154	0.0738
	beta-sitosterol	0.74↓	0.0246	0.1011
	Stigmasterol	1.81↑	0.0424	0.1503
	7-hydroxycholesterol (alpha or beta)	0.42↓	0.0431	0.1503
	glycocholate sulfate	0.22↓	0.0010	0.0101

	Primary Bile Acid Metabolism	Tauro-beta-muricholate	0.52	0.0446	0.1503
	Secondary Bile Acid Metabolism	Taurochenodeoxycholate	0.05↓	0.0001	0.0017
		Deoxycholate	1.49↑	0.0212	0.0907
		isohyodeoxycholate	1.92↑	0.0060	0.0358
Cofactors and Vitamins	Nicotinate and Nicotinamide Metabolism	quinolinate	3.92↑	0.0001	0.0009
		nicotinate ribonucleoside	10.24↑	0.0104	0.0546
		Nicotinamide	0.52↓	0.0360	0.1342
		1-methylnicotinamide	4.03↑	0.0011	0.0106
		trigonelline (N'-methylnicotinate)	4.00↑	0.0020	0.0169
	Pantothenate and CoA Metabolism	pantetheine	2.50↑	0.0022	0.0181
	Ascorbate and Aldarate Metabolism	oxalate (ethanedioate)	2.57↑	0.0000	0.0000
	Tocopherol Metabolism	delta-tocopherol	0.37↓	0.0000	0.0000
		alpha-tocotrienol	1.59↑	0.0003	0.0039
		gamma-tocotrienol	1.46↑	0.0287	0.1145
		gamma-tocopherol/beta-tocopherol	0.33↓	0.0000	0.0000
	Pterin Metabolism	Pterin	0.69↓	0.0332	0.1259
	Hemoglobin and Porphyrin Metabolism	protoporphyrin IX	1.74↑	0.0243	0.1011
		bilirubin (Z,Z)	0.20↓	0.0006	0.0069
		bilirubin (E,E)*	0.20↓	0.0000	0.0006
		biliverdin	0.36↓	0.0121	0.0629
	Thiamin Metabolism	Thiamin (Vitamin B1)	1.77↑	0.0035	0.0245
	Vitamin A Metabolism	Retinol (Vitamin A)	2.42↑	0.0043	0.0285
		Carotene diol (1)	3.04↑	0.0001	0.0016
	Vitamin B6 Metabolism	Pyridoxine (Vitamin B6)	41.89↑	0.0000	0.0000
		Pyridoxal	1.45↑	0.0014	0.0128
		Pyridoxate	2.07↑	0.0007	0.0081
		Pyridoxamine	1.67↑	0.0147	0.0722
Xenobiotics	Benzoate Metabolism	2-hydroxyhippurate (salicylurate)	65.75↑	0.0005	0.0058
		Phenylpropionylglycine	10.2↑	0.0141	0.0706
		3-(3-hydroxyphenyl)propionate sulfate	38.24	0.0033	0.0240
		3-(3-hydroxyphenyl)propionate	19.12↑	0.0000	0.0000

	3-phenylpropionate (hydrocinnamate)	12.38↑	0.0000	0.0000
Xanthine Metabolism	1-methylurate	0.61↓	0.0431	0.1503
	Cinnamoylglycine	13.21↑	0.0242	0.1011
	2-oxindole-3-acetate	7.51↑	0.0000	0.0000
	dihydroferulic acid	6.66↑	0.0000	0.0000
	enterolactone	3.27↑	0.0005	0.0056
	Ferulate	27.13↑	0.0000	0.0000
Food component/Plant	Ferulic Acid 4-sulfate	24.21↑	0.0291	0.1152
	Pheophytin A	37.25↑	0.0000	0.0000
	Pinosylvin	3.36↑	0.0015	0.0132
	Sinapate	4.99↑	0.0000	0.0000
	Tartarate	4.44↑	0.0436	0.1503
	4-hydroxycinnamate	4.66↑	0.0001	0.0012
Drug – Tropical Agent	Salicylate	6.23↑	0.0000	0.0000
Chemical	Thioproline	0.61↓	0.0091	0.0501

Table S9. P-values and q-values for changes in metabolites in human Beans/Bran Enriching Nutritional Eating For Intestinal health Trial (BENEFIT) study rice bran group at 4 weeks compared to baseline and in AOM/DSS mice in the rice bran group at 6, 10, and 14 weeks and baseline. Metabolites listed in the table had a significant fold change in the BENEFIT trial (p <0.05), and a significant fold change in at least 2 time points in the AOM/DSS mice and were not significantly different from control at baseline (p<0.05).

Metabolic Pathway	Metabolite	BENEFIT (4 Weeks/ Baseline)		Mice (2 Weeks/ Baseline)		Mice (6 Weeks/ 2 Weeks)		Mice (10 Weeks/ 6 Weeks)		Mice (14 Weeks/ 10 Weeks)	
		p-value	q-value	p-value	q-value	p-value	q-value	p-value	q-value	p-value	q-value
Histidine Metabolism	N-acetylhistamine	0.0367	0.4121	0.0000	0.0000	0.0001	0.0024	0.9489	0.9347	0.0347	0.1571
Leucine, Isoleucine and Valine Metabolism	Beta-hydroxyisovalerate	0.033	0.4103	0.0000	0.0000	0.0021	0.0123	0.9848	0.9430	0.0328	0.1543
	Ethylmalonate	0.0475	0.4259	0.0011	0.0012	0.5644	0.3183	0.7303	0.9162	0.0362	0.1607
Methionine, Cysteine, SAM and Taurine Metabolism	N-acetylmethionine sulfoxide	0.0043	0.1944	0.0017	0.0019	0.0935	0.1055	0.4420	0.9162	0.0400	0.1741
Gamma-glutamyl Amino Acid	Gamma-glutamylphenylalanine	0.0428	0.4185	0.0007	0.0009	0.1680	0.1557	0.2267	0.8594	0.0046	0.0530
Benzoate Metabolism	p-cresol sulfate	0.0376	0.4156	0.0055	0.0050	0.0481	0.0706	0.3055	0.9061	0.4074	0.4803
Food Component/Plant	Apigenin	0.0002	0.0494	0.0365	0.0256	0.0072	0.0222	0.0539	0.7038	0.0000	0.0000
	Enterolactone	0.0015	0.1027	0.0000	0.0000	0.0280	0.0518	0.3739	0.9162	0.4423	0.4880
